# Fighting Against Bacterial Lipopolysaccharide-Caused Infections through Molecular Dynamics Simulations: A Review

**DOI:** 10.1021/acs.jcim.1c00613

**Published:** 2021-09-24

**Authors:** Cristina González-Fernández, Arantza Basauri, Marcos Fallanza, Eugenio Bringas, Chris Oostenbrink, Inmaculada Ortiz

**Affiliations:** † Department of Chemical and Biomolecular Engineering, ETSIIT, University of Cantabria, Avda. Los Castros s/n, 39005 Santander, Spain; ‡ Institute for Molecular Modeling and Simulation, BOKU − University of Natural Resources and Life Sciences, Muthgasse 18, 1190 Vienna, Austria

**Keywords:** Lipopolysaccharide, molecular dynamics simulations, atomistic resolution, coarse-grained resolution, enhanced sampling, free energy calculation, bacterial infections, multidrug resistance, Gram-negative bacteria

## Abstract

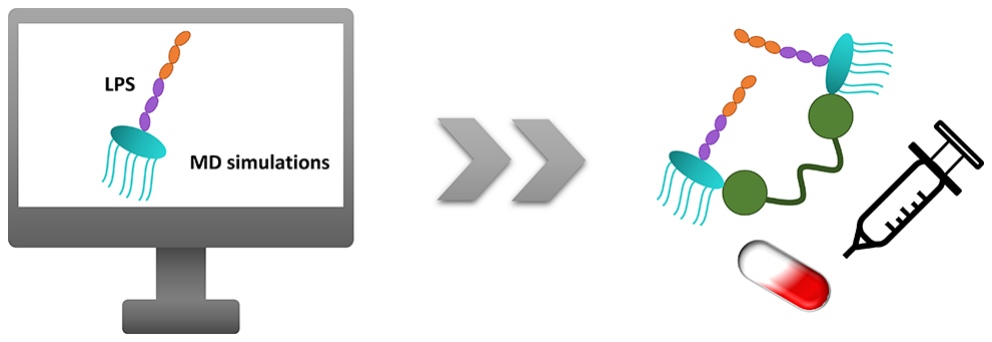

Lipopolysaccharide (LPS) is the primary component of the outer leaflet of Gram-negative bacterial outer membranes. LPS elicits an overwhelming immune response during infection, which can lead to life-threatening sepsis or septic shock for which no suitable treatment is available so far. As a result of the worldwide expanding multidrug-resistant bacteria, the occurrence and frequency of sepsis are expected to increase; thus, there is an urge to develop novel strategies for treating bacterial infections. In this regard, gaining an in-depth understanding about the ability of LPS to both stimulate the host immune system and interact with several molecules is crucial for fighting against LPS-caused infections and allowing for the rational design of novel antisepsis drugs, vaccines and LPS sequestration and detection methods. Molecular dynamics (MD) simulations, which are understood as being a computational microscope, have proven to be of significant value to understand LPS-related phenomena, driving and optimizing experimental research studies. In this work, a comprehensive review on the methods that can be combined with MD simulations, recently applied in LPS research, is provided. We focus especially on both enhanced sampling methods, which enable the exploration of more complex systems and access to larger time scales, and free energy calculation approaches. Thereby, apart from outlining several strategies for surmounting LPS-caused infections, this work reports the current state-of-the-art of the methods applied with MD simulations for moving a step forward in the development of such strategies.

## Introduction

**Figure 1:**
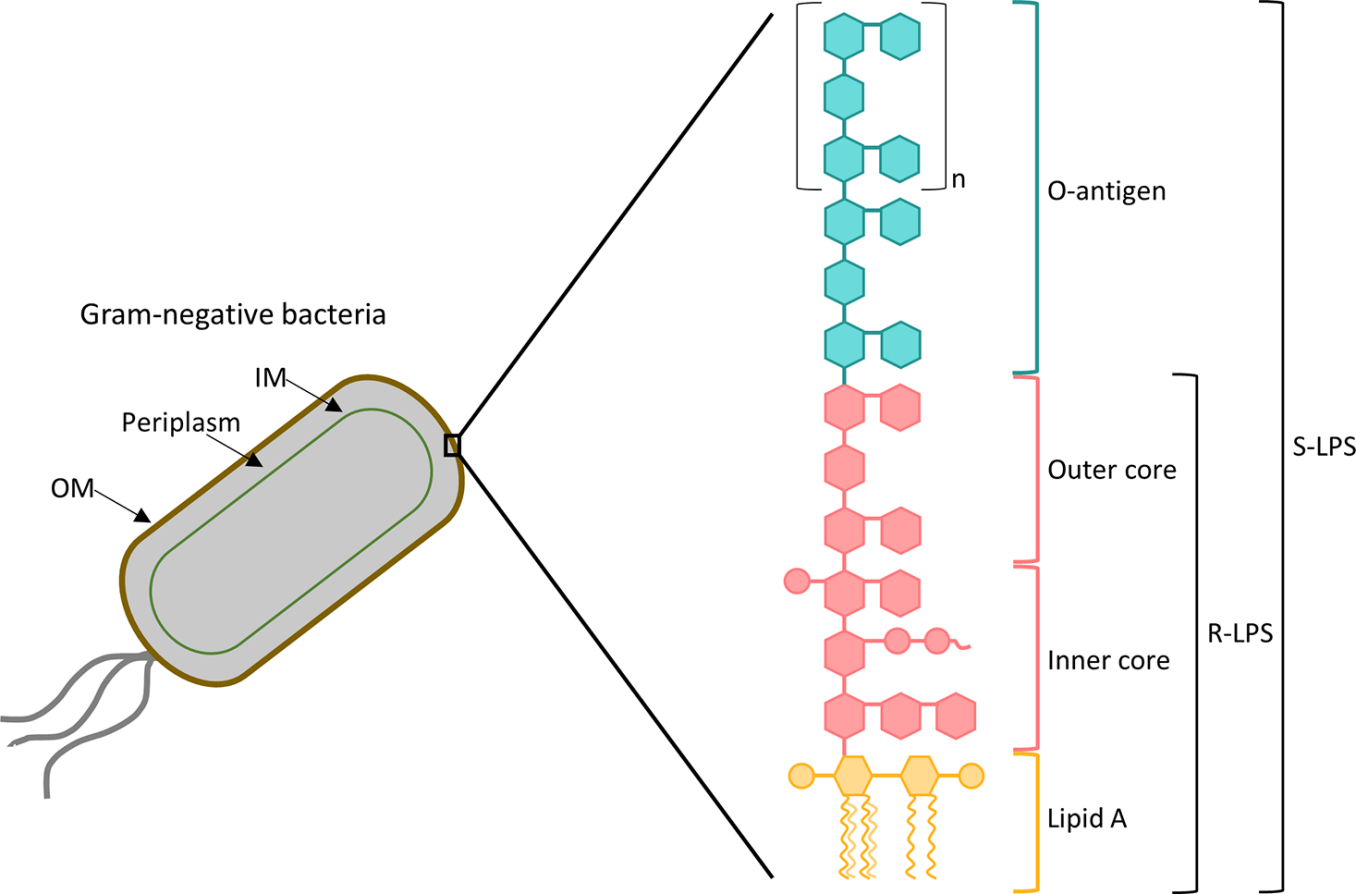
Cell envelop architecture and structure of the LPS of Gram-negative bacteria.

Infections caused by multidrug-resistant bacteria are recognized as one of the greatest threats to public health globally. Specifically, Gram-negative bacteria are more prone to confer resistance to antibiotics than their Gram-positive counterparts, due to the complexity of their layered outer membrane architecture.^1−5^ Thereby, contrary to Gram-positive bacteria, the cell envelope of Gram-negative bacteria is composed of two membranes, which differ in their structure and composition; these membranes are separated by the periplasm, an aqueous compartment that includes a peptidoglycan cell wall (Figure 1).^6−10^ The inner membrane (IM) is a symmetric phospholipid bilayer.^6,8,11^ Conversely, the outer membrane (OM), which represents the first line of defense from environmental threats in Gram-negative bacteria, is asymmetric; thus, the inner leaflet has the same phospholipid composition as the IM, whereas the outer leaflet is mainly composed of lipopolysaccharide (LPS) molecules.^7−9,11^ LPS is the major constituent of the Gram-negative bacterial OM and plays a pivotal role in antibiotic resistance.^12,13^ The structure of LPS comprises three covalently attached domains, namely, the lipophilic lipid A, the hydrophilic core oligosaccharide, and the hydrophilic O-antigen, as schematized in Figure 1.^14−16^ LPS molecules that include these three regions are named as smooth (S-LPS), whereas when the O-antigen and/or portions of the core oligosaccharide are absent, LPS is referred to as rough (R-LPS).^10,15^ The lipid A moiety is the most conserved portion and also the main toxic constituent of LPS;^8,13,15,17^ it has a glucosamine disaccharide backbone that is acylated with varying numbers of acyl chains (from four to eight) and commonly phosphorylated.^6,8,15,18^ The core oligosaccharide presents a relatively conserved structure, where two regions can be distinguished: an inner core proximal to lipid A, that is made of both at least one residue of 3-deoxy-d-manno-oct-2-ulosonic acid (KDO) and several heptoses, and an outer hexose core distal to lipid A.^8,10,15,19^ Finally, the O-antigen consists of oligosaccharide repeating units (up to 40) each having 3–8 sugar residues; it is the most variable constituent of LPS and determines the serological specificity.^10,11,15,19,20^ While lipid A is embedded in the outer leaflet of the bacterial OM and acts as an anchor of LPS to the OM, both the core oligosaccharide and the O-antigen are extended outward.^8,14,15^

LPS is a potent stimulator of the host immune system. However, dysregulation of the host response to bacterial infection could result in life-threatening sepsis and septic shock for which there are no appropriate treatments so far. Hence, since LPS can elicit an immune response toxic for the host, it has been extensively named as an endotoxin.^7,17,21−24^ As a result of the worldwide challenge of multidrug resistance, the occurrence and frequency of sepsis will predictably increase;^24^ thus, the exploitation of novel approaches for treating bacterial infections is urgently needed. In this sense, gaining an in-depth understanding about the LPS–host interactions that take place during immunostimulation, the interaction of LPS with several affinity ligands, and the LPS conformation and dynamics is crucial for making progress on the fight against LPS-caused infections by rationally designing novel antisepsis drugs, vaccines, and LPS detection and sequestration therapeutic strategies. In order to address these tactics, understanding microscopic details of the LPS systems is imperative.

**Figure 2:**
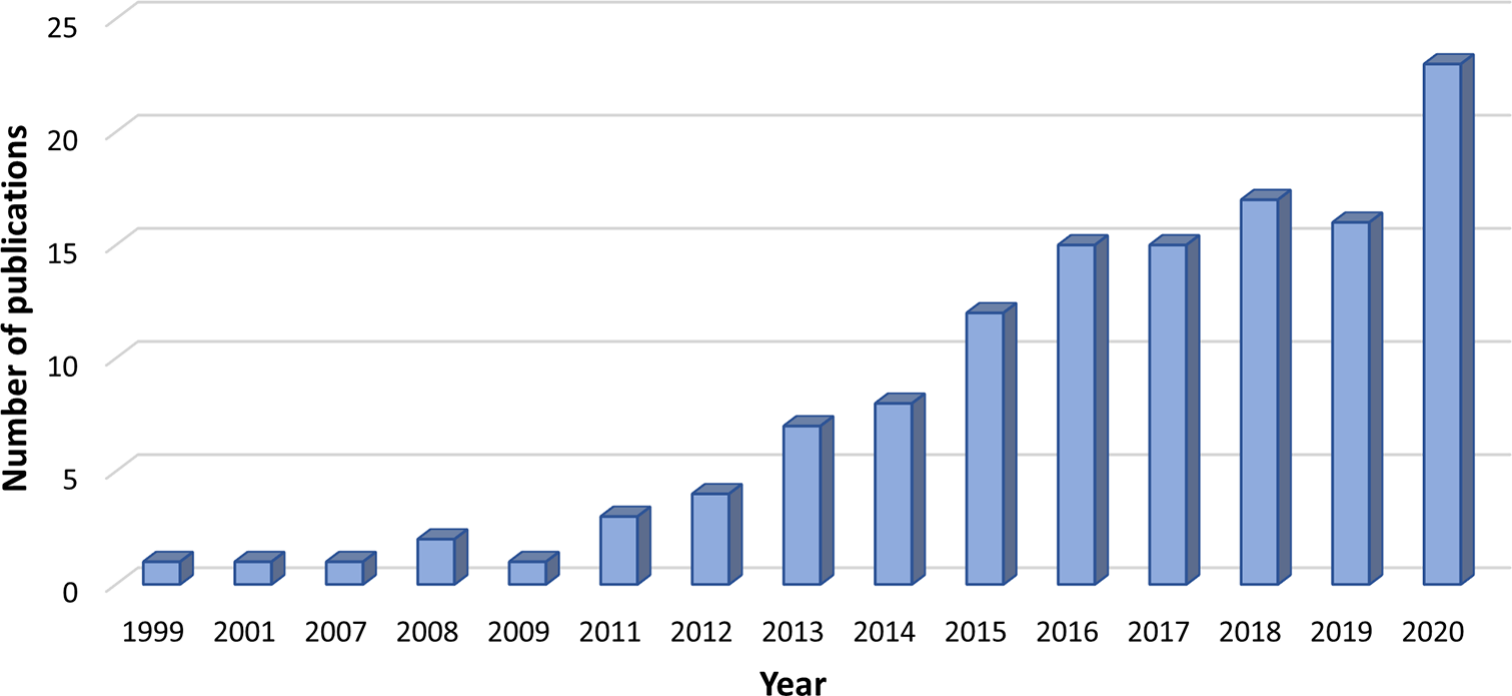
Number of publications in the last 21 years related to the use of MD simulations in LPS research and found in the Scopus database using the restrictive keywords “molecular dynamics simulation*” and “LPS”.

Classical molecular dynamics (MD) simulations have proven valuable for elucidating and understanding the structure, function, and dynamics of LPS as well as its interactions with other molecules at the atomic level. Since MD provides incredibly detailed insights into the molecular process of interest that often goes beyond the reach of sophisticated wet-lab experiments, this *in silico* method facilitates the interpretation of experimental data and can be used as a prior stage to experiments, thus leading to time and cost savings due to the significant minimization of the number of experiments that need to be carried out.^25−31^ The great significance of MD for moving a step forward on the LPS research is supported by the exponential increase of the number of published studies that make use of MD to simulate LPS systems since the late 1990s, as noticed from Figure 2. Such an increase has been fueled by the feasibility of access to biologically meaningful time and length scales, despite the complexity of LPS-related systems, which can be accomplished by combining different computational methods with MD simulations.^26−28,32−35^

In this Review, the computational methods available in the MD field that have been applied to investigate LPS-related phenomena in order to support the fight against LPS-caused infections through several tactics, such as the development of novel antisepsis drugs, vaccines, and endotoxin capturing and detection strategies, are outlined. The impact of these computational methods on the progress of LPS research is also emphasized. We begin with a brief description of both the alternatives for representing molecules in MD simulations and the most important enhanced sampling and free energy calculation methods, highlighting their strengths and critical aspects. Subsequently, studies published in the last four years that explore, using MD simulations, the immunostimulatory ability of LPS, the interactions that endotoxins can establish with several molecules, and the conformation and dynamics of LPS are reported; hence, an overview of the latest advances in LPS research is provided. Furthermore, the applicability and importance of the aforementioned methods to address such studies are discussed. Thereby, this work proves particularly useful not only for gaining insights into several approaches that are currently being investigated for surmounting infections caused by bacterial LPS but also for rationalizing the methods that can be combined with MD simulations in order to address such investigations. Additionally, due to the importance of coupling MD simulations with wet-lab experiments to move forward on the development of the above-mentioned strategies for overcoming bacterial infections, we also emphasize how MD simulations can influence the experimental work. Finally, challenges and future directions in this field are also discussed.

## Theoretical Background

In order to investigate events related to LPS, several MD methods have been applied. These methods range from conventional atomistic to enhanced sampling methods. In this subsection, the MD methods that have been employed in the last four years to explore LPS-related systems are briefly described. A more detailed description of these methods can be found elsewhere.^28,36−43^

**Figure 3:**
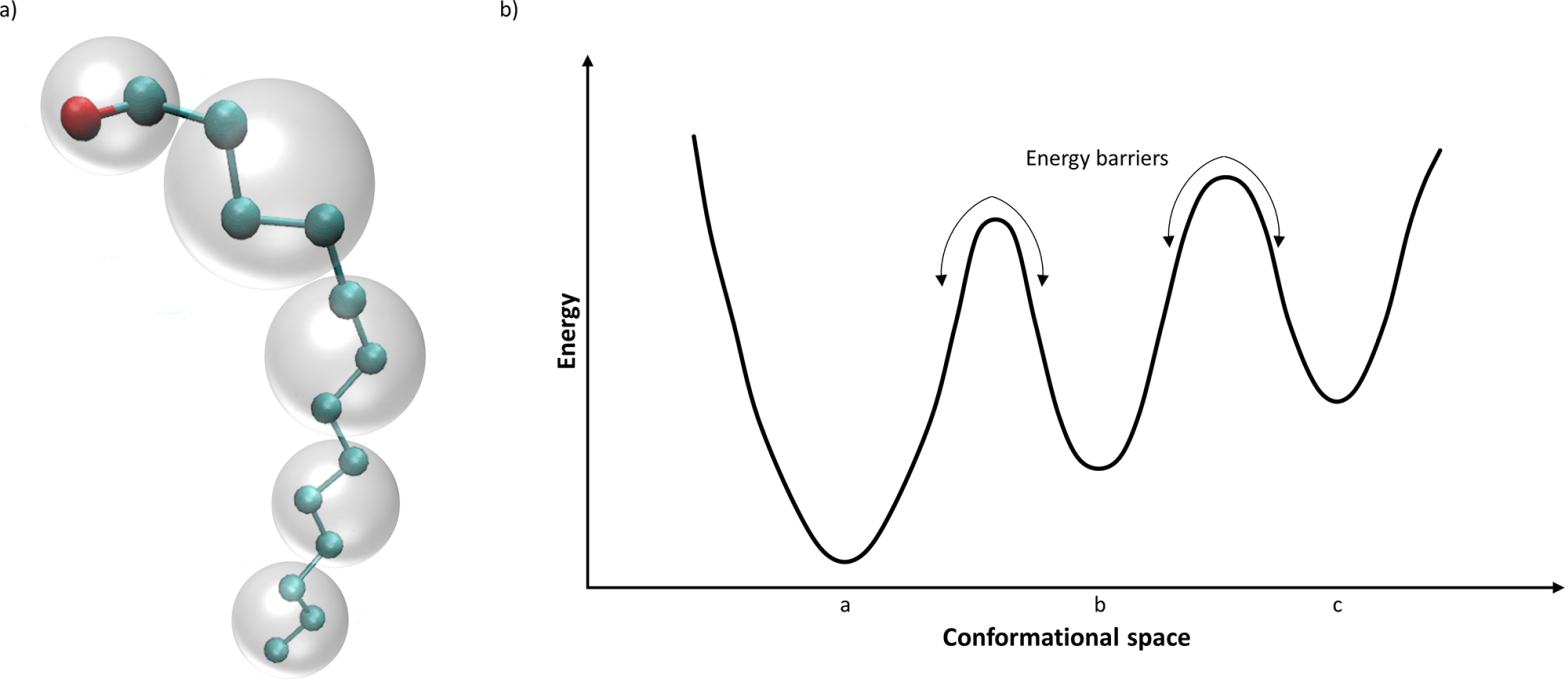
Schematics of (a) all-atom (balls and sticks) and coarse-grained (shaded spheres) representations and (b) the rugged energy landscape of a biomolecule.

Depending on the definition of the elementary particles considered in the model, molecules can be represented at various levels of resolution, as schematized in Figure 3a. All-atom molecular dynamics (AA-MD) simulations, which rely on describing the molecules at atomistic resolution, represent the common approach to reproduce the motion of biomolecular systems.^32−34^ According to this simulation method, the positions and velocities of every atom in the system are determined by solving Newton’s equations.^33,34,44^ Thereby, AA-MD enables the simulation of biological processes of interest with considerable accuracy and a high level of detail.^37,45^ Due to the short time steps of AA-MD simulations (1–2 fs) that are required for ensuring the numerical stability, millions or billions of time steps are typically involved in AA-MD; this fact, along with the millions of interatomic interactions that are commonly evaluated during each time step, makes atomistic simulations greatly computationally demanding.^30,32,46,47^ Therefore, despite enabling the capture of the biomolecules behavior at atomic detail, the time (typically from nanoseconds to microseconds) and length scales that can be achieved by AA-MD simulations are insufficient to explore several biological processes of interest that take place on microsecond to second time scales.^30,33,34,45^

A popular alternative to overcome the limitations of AA-MD is based on simplifying the representation of the biomolecules by using coarse-grained models.^32,33,37^ As such, the modeling of individual atoms, characteristic of AA-MD, is replaced by describing groups of atoms as a single bead, which reduces the number of particle–particle interactions to be computed.^32,33,39,45,48^ Therefore, coarse-grained molecular dynamics (CG-MD) enables the simulation of more complex and larger systems (hundreds of nanometers) and longer time scales (on the order of seconds) since the number of simulated particles is lower.^32,33,39,47,49^ Consequently, as a result of the decrease in the number of degrees of freedom, the potential energy surface is smoother. This fact, along with the absence of high frequency bonds, allows the use of longer integration time steps (10–30 fs) than typical time steps of AA-MD, which in turn implies longer simulations. Besides, smoothing the energy function also leads to faster sampling the conformational states of the system under investigation using CG-MD in comparison to AA-MD over similar time scales.^32,39,46,47^

Although coarse-grained modeling enables the investigation of biomolecular processes beyond the time and length barriers of AA-MD, dismissing atomic details in the models of biomolecules may lead to inaccuracies on the properties to be predicted as well as to the impossibility of examining other properties. Therefore, coupling the accuracy of all-atom models and the efficiency of coarse-grained ones is desirable.^33,37,39,50^ In this regard, atomic resolution and time-size scalability can be accomplished by following strategies such as (i) reconstructing all-atom structures from coarse-grained simulations (known as backmapping or reverse mapping) and (ii) using hybrid all-atom/coarse-grained (AA/CG) models for the simulations.^37,49,51,52^ Backmapping entails the conversion of coarse-grained models to all-atom structures in order to recover atomic information from CG simulations;^49,52^ conversely, hybrid multiscale AA/CG models combine different levels of resolution at once, providing an atomistic description of the regions of interest, while enhancing the sampling speed by using coarse-grained resolution in the remaining regions.^32,43,48,51−53^

Additionally, when performing MD simulations, sufficient sampling of the conformational space of the system under investigation is of paramount importance, so that all physically relevant conformational states can be considered.^54,55^ However, complex biomolecular systems are characterized by rugged energy landscapes, and the crossing of energy barriers between metastable states can be difficult. This fact leads to the trapping of such systems in energy wells of the conformational space, thus hindering the exploration of new states (Figure 3b).^36,41,55,56^ In order to surmount this limitation and widen the sampling time scales that are typically accessed by MD, several enhanced sampling approaches have been developed.^36,56^ Furthermore, an appropriate sampling enables the calculation of the free energy of the processes under study.^32^ Therefore, in the following, we briefly explain the fundamentals of enhanced sampling methods used in LPS research for both exploring slow events and computing the free energy of the phenomena of interest. Prior to such explanation, it should be pointed out that enhanced sampling methods can be applied with both atomistic and coarse-grained molecular representations in order to successfully explore the system of interest.^57^ Additionally, coarse-graining is also an enhanced sampling method since by coarse-graining the system the number of degrees of freedom is reduced and the potential energy surface is smoothened.^32,33^

Umbrella sampling (US) is one of the most commonly used methods for enhancing sampling. In practice, this method introduces a bias potential (termed as umbrella potential) to guide the system from one state to another (e.g., from being free in solution to being bound to a membrane). The pathway between these states is covered by performing independent MD simulations (so-called windows) using umbrella potentials. Subsequently, individual umbrella windows can be combined using different methods, the most commonly used being the weighted histogram analysis method (WHAM). It should be pointed out that the choice of the parameters of the umbrella potentials is of paramount importance; for example, the selection of the force constant is key, since the bias potentials are typically harmonic.^38,41,58−60^ Additionally, Hamiltonian replica-exchange with solute tempering (HREST) has also been used to enhance the sampling efficiency. Particularly, in the HREST2 method, the temperature is the same for all replicas, whereas the potential energy for each of them is scaled differently.^61,62^

On the other hand, in steered MD (SMD) simulations, conformational sampling is facilitated by applying a time-dependent external force to lead the motion of the selected atoms.^36,38,42^ Typically, one end of the molecule is kept fixed, whereas the opposing one is subject to the external force (for instance harmonic), which can be applied following several protocols, including, pulling at constant velocity or force. It is worth mentioning that such force could be exerted to any atom or group of atoms; thus, it is not restricted to be applied to the ends of the molecule.^36,42,63,64^ For this simulation method, the restraint stiffness and the pulling velocity are of paramount importance for the derived results.^36,42^

The free energy of the events under investigation can be computed *in silico* using several methods, which differ in accuracy and computational cost.^32,65,66^ The Linear Interaction Energy (LIE), Molecular Mechanics Poisson–Boltzmann Surface Area (MM-PBSA), and Molecular Mechanics Generalized Born Surface Area (MM-GBSA) methods are frequently used for free energy calculations since they exhibit an intermediate performance in terms of efficiency and accuracy.^65,67,68^ These methods solely evaluate the initial and final states of the system, and thus, they are called end-point methods.^65,67,69^

The LIE method involves performing only two MD simulations: one of the ligand complexed with the receptor and the other of the ligand in solution. Thereby, according to the LIE approach, the binding free energy is linearly proportional to the difference between energy averages of electrostatic and van der Waals interactions of the ligand with its surroundings in the bound and free states; these differences are scaled by two empirical parameters.^38,65,68,70−72^ In the MM-PBSA and MM-GBSA methods, the free energy could be computed from three separate simulations (i.e., ligand–receptor complex, free receptor, and free ligand); however, simulating only the complex is more commonly done due to stability issues.^38,65,68,73^ Hence, the free energy is calculated from the vacuum molecular mechanics (MM) energies, the polar and nonpolar solvation free energies, and the conformational entropy change upon ligand–receptor binding. The polar contribution to the solvation free energy is calculated using the Poisson–Boltzmann equation (MM-PBSA method) or the generalized Born model (MM-GBSA method).^67,68,74,75^

On the other hand, US is one of the most common approaches for calculating the Potential of Mean Force (PMF), i.e., the free energy profile of the event under investigation along a reaction coordinate. To this end, the initial conformations for USMD are commonly provided by other enhanced sampling methods, such as SMD; additionally, individual umbrella windows are typically combined using WHAM.^41,60,76,77^

Once the basics about MD methods, regarding system representation, conformational sampling, and free energy calculation approaches, have been introduced, in the following section, we discuss the use of these methods to investigate LPS-related phenomena. Additionally, a brief picture of different strategies to fight against bacterial infections will be given.

## Progress in LPS Research through MD

Investigating the immunostimulatory ability of LPS, the interactions it can establish with other molecules as well as its conformation and dynamics is of paramount importance in order to surmount the multidrug resistance challenge and combat the Gram-negative bacterial infections by developing novel antisepsis drugs, vaccines, and LPS detection and sequestration therapeutic strategies. MD simulations have been understood as a powerful tool for addressing such issues; particularly, advances in algorithms and computational resources have enabled the study of LPS events at biologically relevant time and length scales. Additionally, the success of MD simulations for examining LPS phenomena has also been fostered by the availability of X-ray crystallographic structures of both LPS and the molecules involved in the events under investigation.^34,37^ In the following subsections, the MD methods that have been used for exploring events related to the immunostimulatory capacity of LPS, its conformation and dynamics, and the interaction of LPS with other molecules are discussed; moreover, the structures employed in these studies that are stored in different databases have been included in Table 1, due to their importance for performing MD simulations. It should be pointed out that studies that are not mentioned in Table 1 typically use modeled structures derived from a variety of methods.

**Table 1:**
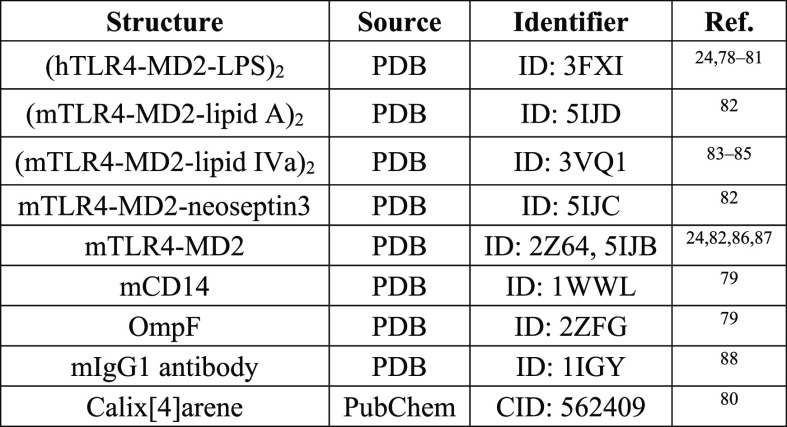
X-ray Crystallographic Structures Stored in the Databases of the Molecules Involved in This Work^a^

a Note: The subindex “2” indicates tetrameric structures.

### LPS Immunostimulatory Ability

**Figure 4:**
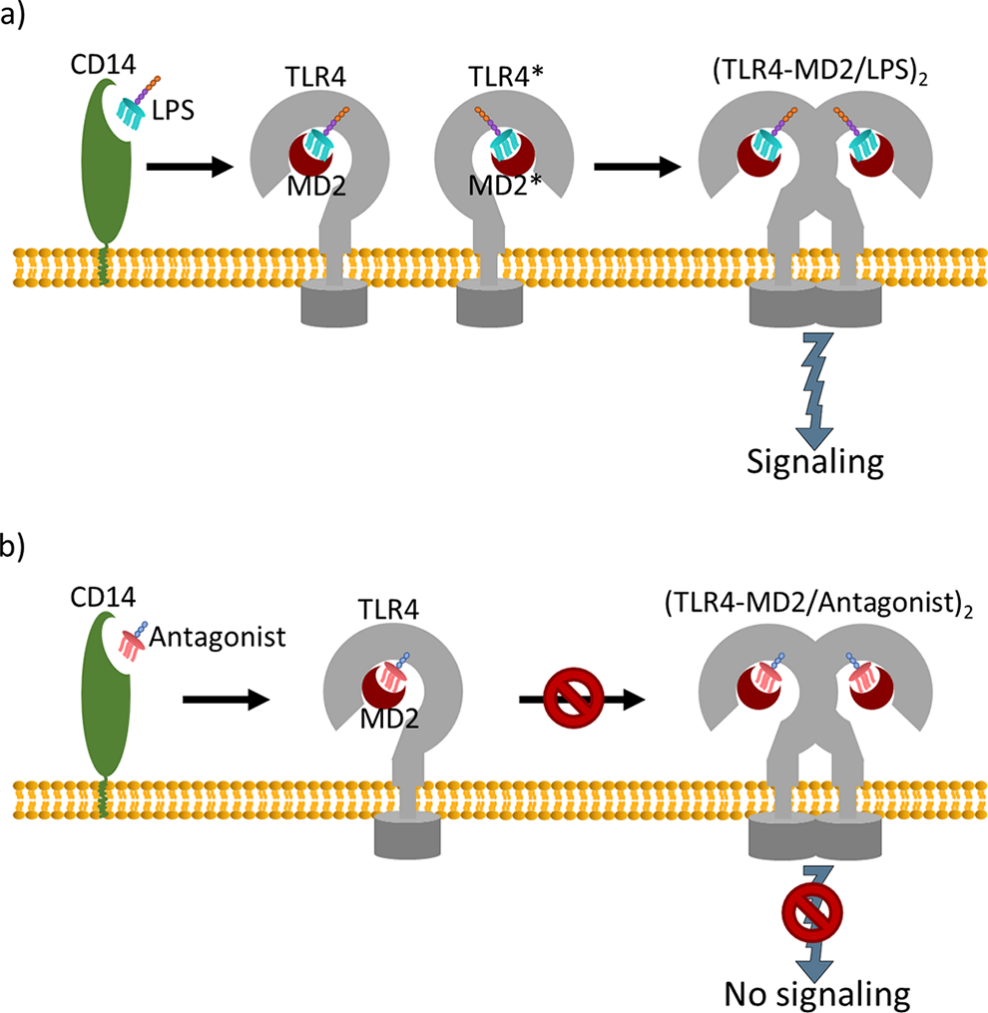
Overview of the activation (a) and inhibition (b) of TLR4–MD2 by LPS and antagonistic molecules, respectively.

LPS molecules can be recognized by the innate immune system, which makes LPS a pathogen associated molecular pattern (PAMP). Thereby, upon bacterial infection, LPS is recognized by the complex composed of Toll-like receptor 4 (TLR4) and myeloid differentiation factor 2 (MD2); as a result of the LPS recognition, the TLR4–MD2 complex triggers a pro-inflammatory response in order to provide an immediate host defense against invading bacteria.^7,17,22,24,79,81,89^ This immune response is advantageous for eliminating bacteria as long as it is controlled. However, the overstimulation of the TLR4-signaling pathway can lead to sepsis and septic shock, which substantiates the endotoxic potential of LPS; specifically, the lipid A moiety of LPS is responsible for such endotoxic activity. Therefore, the development of strategies to diminish the exaggerated and detrimental LPS-induced immune response is of outstanding importance.^21,22,24,79,81,82,86,87,90,91^ In this regard, several MD studies have focused on gaining an improved understanding about key steps in the TLR4 activation by LPS. Moreover, the rational design of TLR4–MD2 antagonists that inhibit TLR4 signaling by competing with LPS in the binding to MD2, as illustrated in Figure 4, has evolved into a hot research topic. Thereby, MD2 and TLR4 have been understood as promising targets for the design of antisepsis drugs, and thus, several TLR4–MD2 inhibitors have been reported in the literature.^22,24,79,82,87,92^ In this subsection, works that make use of MD simulations for moving a step forward in the elucidation of key steps of the TLR4 pathway and in the design of TLR4–MD2 antagonists are discussed; these studies have been included in Table 2.

**Table 2:**
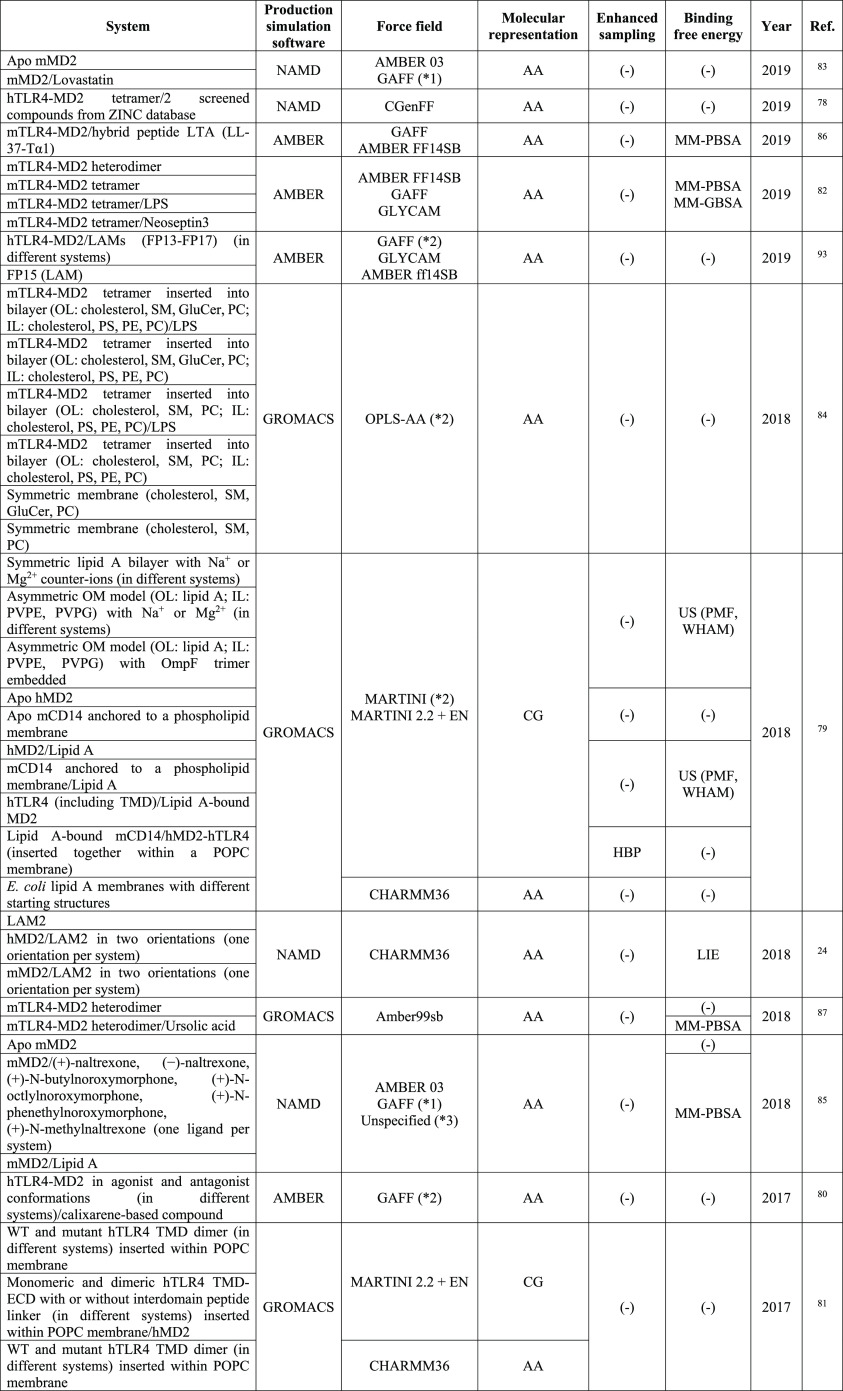
MD Studies Focused on the Immunostimulatory Ability of LPS^a^

a Force fields that have been used for each investigated system have not been specified; force fields that are included in the Table refer to the ones used in the study. *1, GAFF + R.E.D.; *2, specific modifications to the force field were included, check the original publication; *3, the force field for some of the molecules could not be specified in the original publication.

Kargas et al.^81^ employed a multiscale MD simulation approach to elucidate the mechanism and the structural basis underlying the homodimerization of the TLR4 transmembrane domain (TMD), which is a key step in the TLR4 signaling pathway. Hence, CG-MD simulations of the TLR4 TMD embedded in a palmitoyl-oleoyl-phosphatidyl-choline (POPC) membrane were first performed; subsequently, the principal conformations of the TLR4 TMDs assembly were back-mapped to all-atom representations, and AA-MD simulations were carried out. The use of this multiscale approach allowed them to increase the simulation time scale; specifically, they reached simulation times higher than 13 and 100 μs for all-atom and coarse-grained simulations, respectively. Additionally, Kargas et al.^81^ explored the coupling between the ectodomain (ECD) and the TMD of TLR4 in order to rationalize experimental insights previously derived. They carried out CG-MD simulations of monomeric and dimeric ECD/TMD of TLR4 in complex with MD2 within a POPC membrane in the absence or presence of peptide linkers. Further directions of the investigations regarding the domains coupling could be focused on performing similar studies including the cytosolic Toll/interleukin-1 receptor (TIR) domain of the TLR4 and also on refining the molecules’ resolution and, thus, enhancing the accuracy of the simulations using all-atoms models, which entails a considerable increase in the computational demand.

Additionally, Huber et al.^79^ investigated the transfer of the LPS in the TLR4 pathway in near-atomic detail. They developed CG models for lipid A, bacterial OMs, and the individual receptors involved in the TLR4 pathway, namely, CD14 (cluster of differentiation 14), MD2, and TLR4. Specifically, they first tested, through CG-MD simulations, the reliability of the models in order to accurately reproduce atomistic MD simulations or experimental observations. Afterward, they substantiated the hypothesis that lipid A follows a funnel-like transfer through the receptors that comprise the TLR4 cascade. To this end, they performed US and PMF calculations to derive the affinity of lipid A to the immune receptors as well as the energy required for its removal from different membranes (symmetric lipid A bilayer and asymmetric OMs with and without porins) into solvent. Hence, Huber et al.^79^ concluded that the transfer process is promoted by an affinity gradient for lipid A, since the relative binding affinities increased from lipid A aggregates or bacterial OMs without or with inserted porins via CD14 to the terminal TLR4–MD2 complex. Finally, they performed CG-MD simulations of a system composed of lipid A bound to CD14 and the TLR4–MD2 complex at the plasma membrane in order to derive the spontaneous assembly of the CD14–MD2–TLR4 receptors. The resultant conformations were used to simulate the complete transfer of lipid A from CD14 to the TLR4–MD2 complex; for that purpose, they applied a harmonic biased potential in order to promote the lipid A transfer from CD14 to MD2. From these simulations, they proposed a stepwise process for the lipid A exchange between CD14 and TLR4–MD2, which entails the formation of a hydrophobic bridge between CD14 and TLR4–MD2 and the gradual migration of lipid tails from CD14 to MD2.

On the other hand, several potent inhibitors of the TLR4–MD2 activity, such as ursolic acid, have been reported in the literature; however, their inhibition mechanism was poorly understood at atomic detail.^87^ Motivated by this fact, Niu et al.^87^ investigated the inhibition mechanism of the TLR4–MD2 complex by ursolic acid through AA-MD simulations and the MM-PBSA method for estimating the binding free energy. Apart from elucidating the binding mode of ursolic acid with TLR4–MD2, they identified residues that play a pivotal role in the complexation of ursolic acid with TLR4–MD2 by decomposing the binding free energy into the residues’ contribution. From this study, they proposed a possible inhibition mechanism of ursolic acid to TLR4–MD2. Additionally, Tafazzol and Duan^82^ investigated the interactions between the TLR4–MD2 receptor and two ligands, namely, LPS and neoseptin3 (peptidomimetic compound), in order to elucidate the mechanism that underlies the modulation of TLR4–MD2 at atomic detail. To this end, they carried out AA-MD simulations of ligand-bound and ligand-free mouse TLR4–MD2 (mTLR4–MD2) tetramers and a ligand-free mTLR4–MD2 heterodimer. Furthermore, they computed the binding free energy of the dimer interfaces between the monomers in the (TLR4–MD2)_2_ tetramer as well as that of the interface between the heterodimers (TLR4–MD2/TLR4*-MD2*) using the MM-GBSA and MM-PBSA methods. Finally, they identified crucial residues of these interfaces in the formation of the TLR4–MD2 tetramer by performing a per-residue decomposition of the binding free energies.

Furthermore, inhibitors for the TLR4–MD2 complex have been designed.^22,24,92^ For instance, Borio et al.^24^ developed novel TLR4–MD2 antagonists based on anionic glycolipids. In order to elucidate the structural basis for the interaction affinity between such Lipid A mimetics (LAMs) and MD2, they complemented their experimental studies with MD simulations. Hence, they carried out AA-MD simulations of human and mouse MD2 (hMD2 and mMD2, respectively) complexed with the compound with the highest antagonist activity (denoted as LAM2); they computed the antagonist-hMD2/mMD2 binding free energy using the LIE method, and from this calculation, they estimated the LAM2–hMD2 dissociation constant. From the MD studies, they concluded that the binding of LAM2 to hMD2 is 3-fold tighter than to lipid A, which substantiate the antagonist potential of LAM2 and thus their capability for competing with lipid A and displacing it from the binding cleft of hMD2.

Collectively, the complexity of the innate immune receptors requires the use of CG models or the introduction of multiscale approaches (i.e., combination of AA and CG-MD simulations) in order to comprehensively investigate key steps in the TLR4 signaling pathway. Additionally, free energy calculations enable not only the determination of the antagonist potential of TLR4–MD2 inhibitors but also the identification of key residues for the binding of agonistic or antagonistic molecules to the TLR4–MD2 complex as well as the estimation of the energy associated with crucial stages in the TLR4 signaling pathway. Thereby, from MD simulations, the dimerization of TLR4 TMDs and the coupling between the transmembrane and ecto-domains of TLR4 have been investigated; moreover, important interactions for the formation of the TLR4–MD2 tetramer have been identified. Furthermore, it was demonstrated that the stepwise transfer of LPS in the TLR4 pathway is fostered by an affinity gradient. Lastly, the inhibition of the TLR4–MD2 activity by natural (ursolic acid) and synthetic (LAMs) compounds has been computationally explored, revealing the antagonist potential of these molecules.

### LPS as Target Molecule

**Figure 5:**
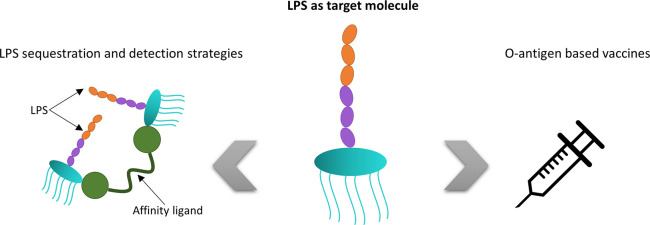
Schematic of the strategies, based on considering LPS as a target, for treating bacterial infections. (Images were freely provided by Pixabay^101^ or created).

The design of novel drugs to downregulate the immune response that LPS induces has received significant attention for treating Gram-negative bacterial infections. However, other strategies are focused on the development of vaccines based on O-antigens. The extracorporeal capture of the LPS released into the bloodstream during infection has also been the focus of intense research; additionally, the development of point-of-care (POC) systems for endotoxin detection in order to diagnose early stage Gram-negative bacterial infections is an unfulfilled need. Therefore, LPS is understood as a target molecule of outstanding interest.^20,91,94−97^ These tactics for fighting against LPS-caused infections have been schematized in Figure 5, and MD studies that address such investigations have been reviewed in Table 3.

**Table 3:**
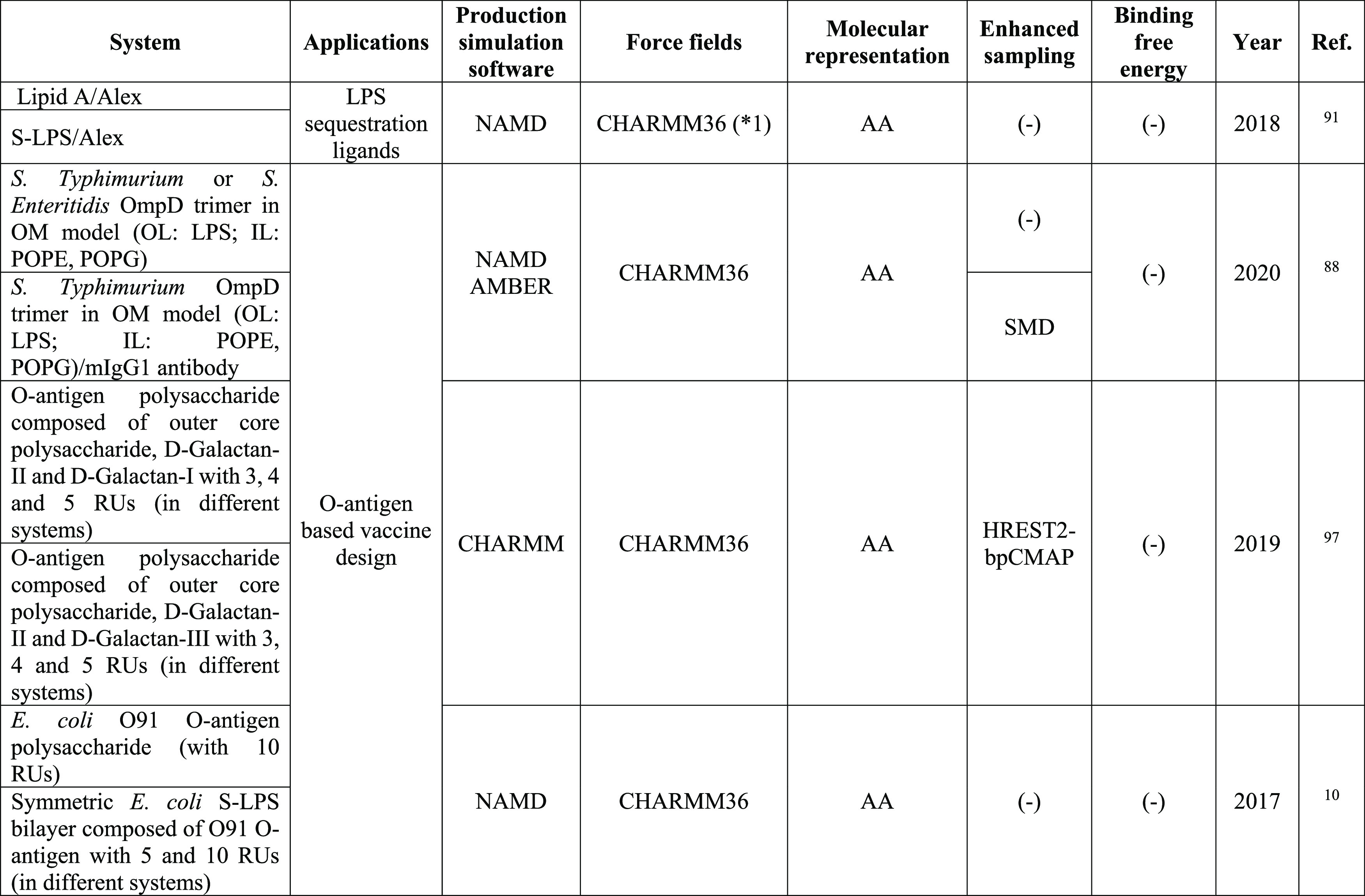
MD Studies Based on LPS as a Target Molecule

*1, specific modifications to the force field were included, check the original publication.

LPS sequestration and detection require the identification of molecules that bind to LPS with high affinity and selectivity, making LPS a target molecule of significant interest. Once LPS binding molecules are found, modifications can be introduced to such ligands in order to enhance the specificity and strength of their interaction with LPS for the specific application.^20,91^ In this regard, Jagtap et al.^91^ assessed the potential of alexidine dihydrochloride (hereafter alex) as an efficient LPS binder in order to be further used for diagnostic purposes. In their study, Jagtap et al.^91^ combined spectroscopy techniques and AA-MD simulations. While the binding sites and stoichiometry could be determined with some of these analytical techniques, they carried out MD simulations to investigate the mechanism of the alex–lipid A and alex–LPS interaction, thus supporting the results they obtained experimentally. It is worth mentioning that, in the first set of simulations, the lipid A portion of LPS, instead of the whole LPS molecule, was considered. This simplification, which significantly reduces the complexity of the systems under investigation, stems from the important role that lipid A has on ligand–LPS binding as well as from the fact that the ability of LPS to activate the immune system can be mainly attributed to its lipid A constituent.

In addition to alex, other molecules that are able to interact with LPS have also been *in silico* investigated. This is particularly the case of the human antimicrobial peptide (AMP) LL-37, polymyxin B (PMB), or Temporin L (TempL) and its analog Q3K-TempL, whose interaction with LPS/lipid A bilayers has been explored by Martynowycz et al.,^98^ Santos et al.,^99^ and Farrotti et al.,^100^ respectively. Additionally, the binding of lipid A to receptors involved in the immune system response (CD14, MD2, TLR4) has been thoroughly investigated by Taffazol and Duan^82^ and Huber et al.^79^ Given that these biomolecules successfully interact with LPS/lipid A, they could be used as LPS sequestration or detection agents. In fact, some of these molecules have been experimentally demonstrated to exhibit a strong affinity toward LPS as discussed by Basauri et al.,^20^ who comprehensively reviewed various LPS binding molecules with different origins. Moreover, the molecular basis of the LPS or lipid A interactions with the aforementioned molecules derived from these computational studies could pave the way for the design of novel molecules with improved binding affinity toward LPS, thus progressing on the development of novel methods for LPS sequestration or detection.

On the other hand, the development of vaccines based on O-antigen polysaccharides has also been of outstanding interest. For that purpose, understanding how the unit length and sequence diversity (i.e., sugar constituents, presence of branched or unbranched structures, etc.) of O-antigens impact their antigenicity is crucial for making further progress on vaccine design.^10,96,97^ In this regard, Blasco et al.^10^ explored the conformation and dynamics of *Escherichia coli* O91 O-antigen by nuclear magnetic resonance (NMR) experiments and extensive MD simulations. Hence, through AA-MD, they investigated such O-antigen polysaccharide when it was free in solution as well as when it was a component of the LPS in a bilayer. For this latter scenario, membranes containing LPS composed of O-antigen polysaccharides with 5 or 10 repeating units (RUs) were modeled. Additionally, Aytenfisu et al.^97^ investigated how modifications in the composition of *Klebsiella pneumoniae* O1 and O2a O-antigen polysaccharides could affect antigenicity. Particularly, they explored the alterations on the conformational properties and accessibilities of these O-antigen polysaccharides, which were polygalactans, resulting from the addition of a branch to their structure. To this end, they simulated O-antigens composed of d-galactan-II and varying numbers of RUs of d-galactan-I or d-galactan-III (branched variant of d-galactan-I) through AA-MD; in this work, sampling was enhanced by combining HREST2 with correction maps as biasing potentials (bpCMAP), namely, the HREST2-bpCMAP method.

Overall, the interactions derived from MD simulations between LPS or lipid A and the molecules reported throughout this work could serve as the cornerstone to design new ligands that bind to endotoxins with high affinity; hence, these molecules could be used to develop LPS sequestration or detection therapeutic strategies. On the other hand, the exploration of the conformation and dynamics of O-antigens through AA-MD simulations contributes to the elucidation of their antigenicity potential, thus facilitating the design of vaccines. Thereby, the studies of Blasco et al.^10^ and Aytenfisu et al.^97^ promote the development of vaccines based on *E. coli* O-91 and *K. pneumoniae* O1 and O2 O-antigen polysaccharides, respectively. It is worth mentioning, as reported by Aytenfisu et al.,^97^ that sampling the conformations of O-antigen polysaccharides can be successfully enhanced by using, for instance, the HREST2 method.

## Coupling MD Simulations and Experimental Work in LPS Research

As it has been demonstrated through this work, important progress on the development of different strategies (i.e., antisepsis drugs, vaccines, and LPS sequestration and detection methods) for fighting against LPS-caused infections has been accomplished by taking advantage of MD. However, many of the reviewed studies are not purely computational, but they combine MD simulations and wet-lab experiments. This fact stems from the potential of MD simulations for supporting wet-lab experiments, either guiding their performance or facilitating the interpretation of experimental results.^30,31^ In this section, we examine representative studies that combine MD and experiments in order to successfully fulfill the aforementioned strategies, thus highlighting the importance of MD simulations to influence experimental work.

MD is commonly used to understand the molecular basis of experimental observations and to support the experimental results. Thereby, Borio et al.^24^ carried out MD simulations and free energy calculations in order to investigate the structural basis underlying the high affinity binding between LAMs, which had been previously developed, synthesized, and biologically evaluated, and human and mouse MD2. They computationally confirmed the TLR4 antagonist potential of one of these LAMs (named as LAM2), which was in agreement with the results from the *in vitro* experiments. Similarly, Peng and co-workers^83^ performed *in vitro* and *in silico* studies in order to assess the suitability of lovastatin as a TLR4 antagonist. Particularly, they drew on MD simulations to elucidate the interaction of lovastatin and MD2. The findings derived from the simulations regarding the lovastatin binding site in MD2 and the stabilization of the MD2 conformation correlated well with the experimental results. Additionally, the performance of *in vitro* and *in silico* assays allowed Zhang et al.^85^ to examine the molecular recognitions and binding modes of (+)-naltrexone-based TLR4 antagonists and MD2. Particularly, MD simulations were carried out to gain molecular insights and investigate the dynamics of the interaction of (+)-naltrexone, its derivates, and lipid A with MD2; moreover, the computationally calculated binding free energies were in agreement with their TLR4 antagonistic activities and binding affinities determined experimentally. On the other hand, Jagtap et al.^91^ combined several experimental techniques and MD simulations to explore the mechanism of interaction between alex and *E. coli* LPS. Thereby, they experimentally determined the binding stoichiometry, the binding sites, and the thermodynamic and kinetic binding and dissociation constants, whereas MD simulations were carried out to support experimental data and to gain insights into the conformational interaction between these molecules. *In silico* results were in agreement with the experimental ones, which proves the potential of alex to neutralize LPS. Finally, aiming at assessing the self-assembly propensity in solution of LAMs that were designed to be further used as TLR4 modulators, Cochet et al.^93^ performed MD simulations of one of these LAMs in water in order to understand, at an atomistic level, its aggregation behavior that they had observed experimentally.

Additionally, MD simulations can be performed prior to experiments in order to guide their performance. In this regard, Sestito and co-workers^80^ performed MD simulations to assess the binding pose stability of one of the calixarene-based TLR4 antagonists that they had designed with the TLR4–MD2 complex and also to elucidate the interactions that were involved in such binding. Once the calixarene/TLR4-MD2 binding and thus the antagonist potential of such calixarene was *in silico* investigated, these calixarenes were synthesized and their capacity to inhibit LPS-stimulated TLR4 signaling was experimentally assessed. Similarly, Cochet et al.^93^ elucidated the binding interactions between several *in silico* designed LAMs with the TLR4–MD2 complex by MD simulations in order to evaluate their possible applicability as TLR4 modulators. Subsequently, they synthesized these LAMs and tested experimentally their ability to bind to hMD2.

Collectively, MD simulations can be integrated with wet-lab experiments in different ways in order to make progress on the development of strategies for fighting against bacterial infections. Thereby, MD can be used after the experiments in order to support experimental evidence and to facilitate their rationalization due to the atomistic insights that can be derived from this *in silico* method. Additionally, when MD simulations are performed prior to wet-lab experiments, the knowledge derived from MD can be used to guide experimental work. Regardless of how MD is coupled with the experiments, it has demonstrated its potential for influencing experimental work.

## Further Directions and Concluding Remarks

MD simulations have been applied to gain an improved understanding about phenomena related to LPS and, in turn, to develop strategies in order to surmount Gram-negative bacterial infections, as evidenced from previous sections. Throughout this work, it has been indicated that such an investigation has been addressed, in most studies, by combining several computational methods, namely, enhanced sampling and free energy calculation methods, with MD simulations. Particularly, the combination of enhanced sampling methods with MD simulations has made it possible to close the gap between the time and length scales that can be accessed with MD simulations and the ones involved in biologically relevant processes. In this regard, advances on the elucidation of the molecular basis for the activation and inhibition of TLR4–MD2 signaling and the design of vaccines based on O-antigens as well as on the capture of LPS by bioaffinity ligands have been made. These investigations have significantly contributed to the rational development of diagnostic and therapeutic strategies for fighting against LPS-caused sepsis. Thereby, knowledge gained from MD simulations has proved crucial for advancing the development of sepsis treatments, for example, by discovering hit or lead compounds for the design of immunomodulatory and anti-inflammatory molecules or by meeting the need of identifying molecules that could replace some trapping molecules that are currently used for the extracorporeal clearance of LPS from blood due to the associated health hazards they pose, such as PMB. Additionally, MD simulations have played an important role in the development of *in vitro* diagnostic systems for the early detection of bacterial infections, which is key for enhancing survival for LPS patients. However, further progress on the development of these strategies requires a deeper exploration of the TLR4 signaling pathway and the interaction of LPS with different molecules, which calls for the investigation of phenomena whose characteristic time and length scales could still be beyond the possibilities of current day MD simulations. Under this scenario, as it has been demonstrated in this work, the development of multiscale models represents an interesting alternative for moving a step forward in the exploration of different phenomena related to LPS in order to fight against bacterial infections.

Overall, in this broad overview about the application of MD simulations to explore different LPS-related phenomena, complemented with their use in the interpretation of experimental evidence or for guiding the performance of experiments, we provide a global picture about the great importance of MD simulations in the development of strategies for overcoming LPS-caused infections. With a special focus on the combination of MD simulations and several computational methods (i.e., enhanced sampling and free energy calculation approaches), this Review proves the potential of MD to be used as a predictive tool.

## Data and Software Availability

The production simulation software used in the works reviewed throughout this study are free of charge and can be downloaded from their corresponding Web sites: GROMACS (http://www.gromacs.org/), NAMD (http://www.ks.uiuc.edu/Research/namd/), AMBER (https://ambermd.org/), and CHARMM (https://www.charmm.org/). In most of the reviewed works, relevant data are included in the manuscript and/or in the Supporting Information files; in other studies, data are made available upon request.^79,84,88^

## Abbreviations

AA: all-atom
alex: alexidine dihydrochloride
AMP: antimicrobial peptide
bpCMAP: correction maps as biasing potentials
CD14: cluster of differentiation 14
CG: coarse-grained
CGenFF: CHARMM general force field
ECD: ectodomain
*E. coli*: *Escherichia coli*
EN: elastic network
GAFF: general AMBER force field
GluCer: glucosylceramide
HBP: harmonic biasing potential
HREST: Hamiltonian replica-exchange with solute tempering
KDO: 3-deoxy-d-manno-oct-2-ulosonic acid
*K. pneumoniae*: *Klebsiella pneumoniae*
IL: inner leaflet
IM: inner membrane
LAMs: lipid A mimetics
LIE: linear interaction energy
MD: molecular dynamics
MD2: myeloid differentiation factor 2
METH: methamphetamine
MM: molecular mechanics
MM-PBSA: molecular mechanics Poisson–Boltzmann surface area
MM-GBSA: molecular mechanics generalized Born surface area
NMR: nuclear magnetic resonance
LPS: lipopolysaccharide
OM: outer membrane
OL: outer leaflet
PAMP: pathogen associated molecular pattern
PC: phosphatidylcholine
PDB: protein data bank
PE: phosphatidylethanolamine
PMB: polymyxin B
PMF: potential of mean force
POC: point-of-care
POPC: palmitoyl-oleoyl-phosphatidyl-choline
POPE: palmitoyl-oleoyl-phosphatidylethanolamine
POPG: 1-palmitoyl-2-oleoyl-phosphatidylglycerol
PS: phosphatidylserine
PVPE: 1-palmitoyl-2-vacenoyl-phosphatidylethanolamine
PVPG: 1-palmitoyl-2-vacenoyl-phosphatidylglycerol
RUs: repeating units
R-LPS: rough lipopolysaccharide
*S. typhimurium*: *Salmonella typhimurium*
*S. enteritidis*: *Salmonella enteritidis*
SM: sphingomyelin
SMD: steered molecular dynamics
S-LPS: smooth lipopolysaccharide
TempL: temporin L
TIR: Toll/interleukin-1 receptor
TLR4: Toll-like receptor 4
TMD: transmembrane domain
US: umbrella sampling
WHAM: weighted histogram analysis method
WT: wild-type
